# Curcumin Promotes Diabetic Foot Ulcer Wound Healing by Inhibiting miR-152-3p and Activating the FBN1/TGF-β Pathway

**DOI:** 10.1007/s12033-023-01027-z

**Published:** 2024-01-11

**Authors:** Mei Cao, Zhisheng Duan, Xianting Wang, Pan Gong, Limei Zhang, Bin Ruan

**Affiliations:** 1 Endocrinology Department, The Third People’s Hospital of Yunnan Province, Kunming, 650011 Yunnan China; 2 Endocrinology Department, Clinical Medical College of Dali University, Dali, 671000 Yunnan China; 3 Occupational Diseases Department, The Third People’s Hospital of Yunnan Province, No. 292 Beijing Road, Guandu District, Kunming, 650011 Yunnan China

**Keywords:** Diabetic foot ulcer, Curcumin, miR-152-3p, FBN1, TGF-β

## Abstract

The objective of this study was to investigate the mechanism of curcumin in diabetic foot ulcer (DFU) wound healing. A DFU rat model was established, and fibroblasts were cultured in a high-glucose (HG) environment to create a cell model. Various techniques, including Western blot, RT‒qPCR, flow cytometry, Transwell, cell scratch test and H&E staining, were employed to measure the levels of relevant genes and proteins, as well as to assess cell proliferation, apoptosis, migration, and pathological changes. The results showed that miR-152-3p was overexpressed in DFU patients, while FBN1 was underexpressed. Curcumin was found to inhibit fibroblast apoptosis, promote proliferation, migration, and angiogenesis in DFU rats, and accelerate wound healing in DFU rats. In addition, overexpression of miR-152-3p weakened the therapeutic effect of curcumin, while overexpression of FBN1 reversed the effects of the miR-152-3p mimic. Further investigations into the underlying mechanisms revealed that curcumin expedited wound healing in DFU rats by restoring the FBN1/TGF-β pathway through the inhibition of miR-152-3p. In conclusion, curcumin can suppress the activity of miR-152-3p, which, in turn, leads to the rejuvenation of the FBN1/TGF-β pathway and accelerates DFU wound healing.

## Introduction

Diabetes mellitus is a common endocrine and metabolic disease that is characterized by chronic hyperglycemia incurred by insufficient insulin secretion or insulin resistance. It is presumed that by 2035, the number of diabetic patients worldwide will reach 600 million, seriously affecting human health [1]. Diabetic foot ulcers (DFUs) are one of the most common complications of diabetes, and it is estimated that 19–34% of people with diabetes will be impacted by DFU during their lifetime [2]. Once foot ulceration occurs, diabetic patients are more likely to have invasive infections in their limbs, which elevates the risk of amputation [3]. The progression of DFUs is polyfactorial. Diabetic peripheral arterial disease, peripheral deliration and foot malformation are the main causes of DFU [4]. The treatment of DFU is primarily symptomatic, including debridement, treatment with appropriate antibiotics, and promotion of healing [5, 6]. However, the therapeutic effect is not good, and the prognosis has not been observably moderated, which is still an enormous clinical difficulty. Therefore, it is urgent to find drugs to remedy DFU.

Curcumin is a lipophilic polyphenol that has antidiabetic, anti-inflammatory, antiaging and anticancer therapeutic potential, and it is obtained by extraction from *Curcuma longa* [7]. Curcumin has mutual effects with many molecules and cellular targets and regulates gene levels by regulating epigenetic embellishment (i.e., DNA methylation, histone modification, and microRNA expression), thereby affecting multifarious pathways to exert multifarious biological activities [8]. Curcumin has been shown to improve diabetic nephropathy by restraining the NLRP3 inflammasome pathway [9]. Moreover, curcumin can facilitate autophagy to restrain renal tubular epithelial cell apoptosis incurred by advanced glycation end products (AGEs) [10]. In addition, curcumin has also been attested to have prominent wound healing capabilities, acting on all phases of the artificial wound recovery course to facilitate its cure [11]. Although curcumin has been extensively studied as a wound healing agent, its role in DFU wound healing needs further study.

It has been mentioned that curcumin can regulate microRNA (miRNA) expression and then exert multifarious biological functions. MiRNAs, which have a length of approximately 20–24 nt, can regulate target genes by directly combining with the mRNAs of target genes and play a pivotal role in multifarious pathophysiological processes [12], including diabetic wound healing. For example, Zhong et al. [13] Studies have shown that inhibition of miR-133b could restore EGFR expression and accelerate wound healing impeded by diabetes. Wang et al. [14] have shown that decreased expression of miR-138 can accelerate wound healing in DFU rats. Similarly, studies have shown that suppression of miR-152-3p can restrain apoptosis, facilitate fibroblast proliferation and transfer, and ultimately accelerate DFU wound healing [15]. Based on the previous results, this study will continue to reveal the downstream regulatory mechanism of miR-152-3p in the regulation of DFU wound healing.

Fibrillin-1 (FBN1) is the main structural component of extracellular microfibrils, which are diffusely interspersed in miscellaneous tissues and play a pivotal role in sustaining connective tissue structure by participating in the formation of elastic fibers [16]. At present, many studies have shown that FBN1 is interrelated with wound healing and fibroblast proliferation and differentiation [17–19]. For example, Li [20] reported that overexpression of FBN1 can facilitate fibroblast proliferation and transfer and restrain apoptosis, thereby accelerating wound recovery in DFUs. Moreover, FBN1 acts as a structural module that facilitates the extracellular regulation of transforming growth factor-β (TGF-β) storage, release, and activation [21]. Microfiber breakage induced by FBN1 can promote the activation of TGF-β1 [22]. TGF-β is a pleiotropic growth factor that impacts wound healing and incorporates 3 isoforms (TGF-β1, TGF-β2, and TGF-β3) [23]. It is excreted by inflammatory cells such as macrophages and revitalizes potent growth factors that regulate cell proliferation, migration, differentiation, and survival, and its activation is usually tightly regulated [24]. Moreover, epithelial-mesenchymal transition induced by TGF-β is a pivotal inducer of tissue formation and regeneration during wound healing [25]. In addition, TGF-β has been reported to promote wound healing in diabetic patients [26]. These findings demonstrate that FBN1 and TGF-β have important roles in DFU healing. However, there is no report that FBN1 and TGF-β coregulate DFU.

Therefore, this study aims to further explore the molecular mechanism by which curcumin influences the DFU process by regulating the expression of miR-152-3p to provide a theoretical basis for the clinical treatment of DFU.

## Materials and Methods

### Clinical Trial

Venous blood was collected from the elbows of 20 pairs of healthy individuals and DFU patients from the hospital, and informed consent was obtained from all individual participants prior to any procedures related to the study. All the methods/studies were in accordance with the Declaration of Helsinki. The experiment was approved by the Medical Ethics Committee of Dali University.

### Establishment of the DFU Model in Rats

Fifty male Sprague‒Dawley (SD) rats (180–220 g) aged 4–6 weeks were obtained from the Animal Experimental Center of Kunming Medical University. The rats were fed under restrained conditions (22 ± 2 °C, 45% humidity, 12 h light/dark cycle), and the feed was strictly sterilized. After 4–5 d of accommodative feeding, the rats were randomly divided into 5 groups (n = 10). A high-fat diet combined with intraperitoneal injection of streptozotocin (STZ, 50 mg/kg) for 5 consecutive days induced diabetes. The control group was fed a normal diet and given the same amount of sodium citrate buffer. After 7 d, the rats with high blood glucose levels (> 250 mg/dL) were selected as diabetic models. After the diabetic rats were anesthetized with isoflurane, rectangular wounds (2 mm × 5 mm) were made on the right instep surface of each rat to establish a diabetic foot ulcer rat model. The curcumin treatment group was administered 300 mg/kg/day curcumin intragastrically for 12 days. The lentivirus (1 × 10^8^ UT/50 μL, GenePharma) containing miR-152-3p mimic or OE-FBN1 was injected into rats via the tail vein. During treatment, the wounds on the rats' right feet were observed and photographed. All methods were conducted in accordance with ARRIVE guidelines, and all animal experiments were approved by the Animal Ethics Committee of Dali University.

### Isolation and Culture of Fibroblasts

The newborn SD rats were killed, and the back skin was clipped under sterile conditions. The subcutaneous tissue was removed and rinsed with PBS solution repeatedly. Then, the tissue was cut into small pieces of 0.5–1 mm^3^ and treated with 0.25% neutral protease at 4 °C for 12–16 h. The epidermis was removed, and the remaining dermal tissue was placed in a 25 cm culture bottle with a small amount of DMEM culture solution containing 10% FBS and cultured at 37 °C and 5% CO_2_ for 24 h. After the tissue block was adsorbed, the culture solution was added to the normal volume. Then, the medium was replaced every 3–4 days. When the cell confluence reached 80%-90%, cell passage was performed. Generations 3–5 of cells were used for follow-up experiments.

### Construction and Transfection of the Cell Model

Fibroblasts were cultured in vitro with 5.5 mmol/L and 30 mmol/L glucose for 48 h as the normal control group and high glucose group (HG group), respectively. Curcumin (30 μM) was added to the treatment group. The treated fibroblasts were cultured overnight in 6-well plates, and when the cell density reached approximately 60–70%, miR-152-3p mimic and OE-FBN1 (GenePharma, China) were transfected into cells according to the Lipofectamine 3000 reagent (Invitrogen, Grand Island, N.Y., USA) instructions and cultured at 37 °C and 5% CO_2_ for 48 h. The transfection efficiency was then determined and used for subsequent experiments.

### RT‒qPCR

Total RNA was extracted from cells and tissues by TRIzol Reagent. The RNA was reverse transcribed into cDNA using a First-Strand cDNA Synthesis Kit. Real-time PCR was performed with U6 and GAPDH as internal controls, and differential gene expression was analyzed by the 2^−ΔΔCt^ method. The primers are shown in Table 1.

**Table 1:** Primer sequences

Target	Sequence(F: Forward primer; R: Reversed primer) (5´-3´)
has-miR-152-3p	F: ACAGAACGGGCCCGGA R: AGTGCAGGGTCCGAGGTATT
mmu-miR-152-3p	F: CAGCAGGCAGACAGAACGG R: AGTGCAGGGTCCGAGGTATT
FBN1	F: GAGATCGCCCTGGGATTTAC
	R: TGGAGGCATCAGTTTCGTTT
U6	F: CTCGCTTCGGCAGCACA
	R: AACGCTTCACGAATTTGCG
GAPDH	F: GGGAAACTGTGGCGTGAT
	R: AAAGGTGGAGGAGTGGGT

### Western blot

Total proteins were extracted from cells and tissues by RIPA buffer containing 1% protease suppressors and phosphatase suppressors. A BCA assay kit (Solarbio, Beijing, China) was used to detect the protein concentration. Total proteins were isolated by SDS‒PAGE, and the separated proteins were transferred to PVDF membranes and blocked with 5% skim milk powder at room temperature for 1.5 h. Then, the following diluted primary antibodies were added: FBN1 (1:1000, ab231094, Abcam, UK), TGF-β (1:1000, ab215715, Abcam, UK), Bax (1: 1,000, ab32503, Abcam, UK), Bcl-2 (1 ab32124, Abcam, UK), cleaved caspase-3 (1:500, ab32042, Abcam, UK), and β-actin (1:1000, ab8226, Abcam, UK) overnight at 4 °C. Next, the membrane was incubated with secondary antibody (1:4000, ab97051, Abcam, UK) for 1 h at room temperature and developed with an ECL kit. Ultimately, the bands were semiquantitatively analyzed by ImageJ software.

### Cell Viability was Determined by CCK-8

The fibroblasts (5 × 10^3^ cells/well) were seeded in a 96-well plate and cultured at 37 °C in a 5% CO_2_ hatcher for 24 h. The cells of each group were treated according to the group, and 10 μL CCK-8 reagent was added to each well after treatment. After 1 h of hatching, OD values at 450 nm were detected by a microplate reader.

### Apoptosis was Analyzed by Flow Cytometry

The treated cells were collected, washed twice with PBS, and then suspended in 200 μL of PBS. An Annexin-V-FITC/PI apoptosis kit (Absin, China) was used to detect the apoptosis rate. According to the manufacturer's instructions, 5 μL PI and 5 μL Annexin V-FITC were added and incubated in darkness for 15 min, and apoptosis was then detected using flow cytometry.

### Transwell Detection of Cell Migration

The concentration of cells in each group was adjusted to 1 × 10^5^ cells/mL by serum-free DMEM substrate. A 200 μL suspension was added to the upper chamber of the Transwell, and 600 μL of DMEM substrate containing 10% FBS was added to the lower chamber. After culturing for 24 h, the cells were dyed with crystal violet (Solarbio, Beijing, China), the number of cells at the immobilized position in each well was counted under a microscope, and 3 fields of view were selected for counting and photographing.

### Scratch Test

Fibroblasts in the logarithmic growth stage were inoculated on 6-well plates. When the cell density reached 90%, a pipette tip was used to make a scratch. After 0 h and 24 h of cell culture, cell migration was observed under a microscope and photographed.

### Immunohistochemistry

The paraffin-embedded foot tissues were deparaffinized and rehydrated before antigen retrieval in 0.01 M citrate buffer (pH 6.0). Slices were incubated with Ki67 (1:200, ab15580) and K14 (1:200, ab52946) overnight at 4 °C, and HRP-labeled secondary antibody and DAB chromogenic agent were used for immunoassay. The results were observed and photographed under a microscope (Nikon, Japan).

### HE Staining

The foot tissues of rats were extracted and made into paraffin sections, dewaxed and hydrated. Then, the slices were dyed in hematoxylin for 5 min, distinguished with 5% acetic acid for 1 min, treated with reversion blue solution for 1–2 min, stained in eosin solution for 1 min, dehydrated in ethanol, and sealed with neutral gum for observation and analysis.

### Dual-Luciferase Reporter Assay

The biological information network (http://starbase.sysu.edu.cn/) was used to predict miR-152-3p with FBN1 binding sites. The FBN1 3'-UTR containing the miR-152-3p binding site was cloned and inserted into the pGL3 vector (Promega, USA), and the wild-type FBN1 vector (WT) was constructed. The mutant FBN1 vector (MUT) was generated by a site-directed mutagenesis kit. WT or MUT vector was cotransfected into 293 T cells with miR-152-3p mimic or negative control using Lipofectamine 3000. The luciferase activity was detected by a luciferase reporter system (Promega, USA) after 48 h.

### MDA and SOD Detection

According to the instructions of the SOD kit (BC0170, Solarbio, Beijing, China) and the MDA kit (A003-1, Jiancheng, Nanjing, China), the tissue was homogenized in an ice bath at a tissue mass ratio (g): extract volume (mL) of 1:5; 8000 g was centrifuged at 4 ℃ for 10 min, and the supernatant was placed on ice for testing. Ultimately, the contents of SOD and MDA were detected in light of specifications.

### Statistical Analysis

GraphPad Prism 8.0 was used to analyze the experimental data and plot the graphics. All experiments were replicated at least 3 times. One-way ANOVA and t test were used for statistical analysis. P < 0.05 was considered statistically significant.

## Result

### miR-152-3p and FBN1 Were Aberrantly Expressed in DFU Patients

**Fig. 1:**
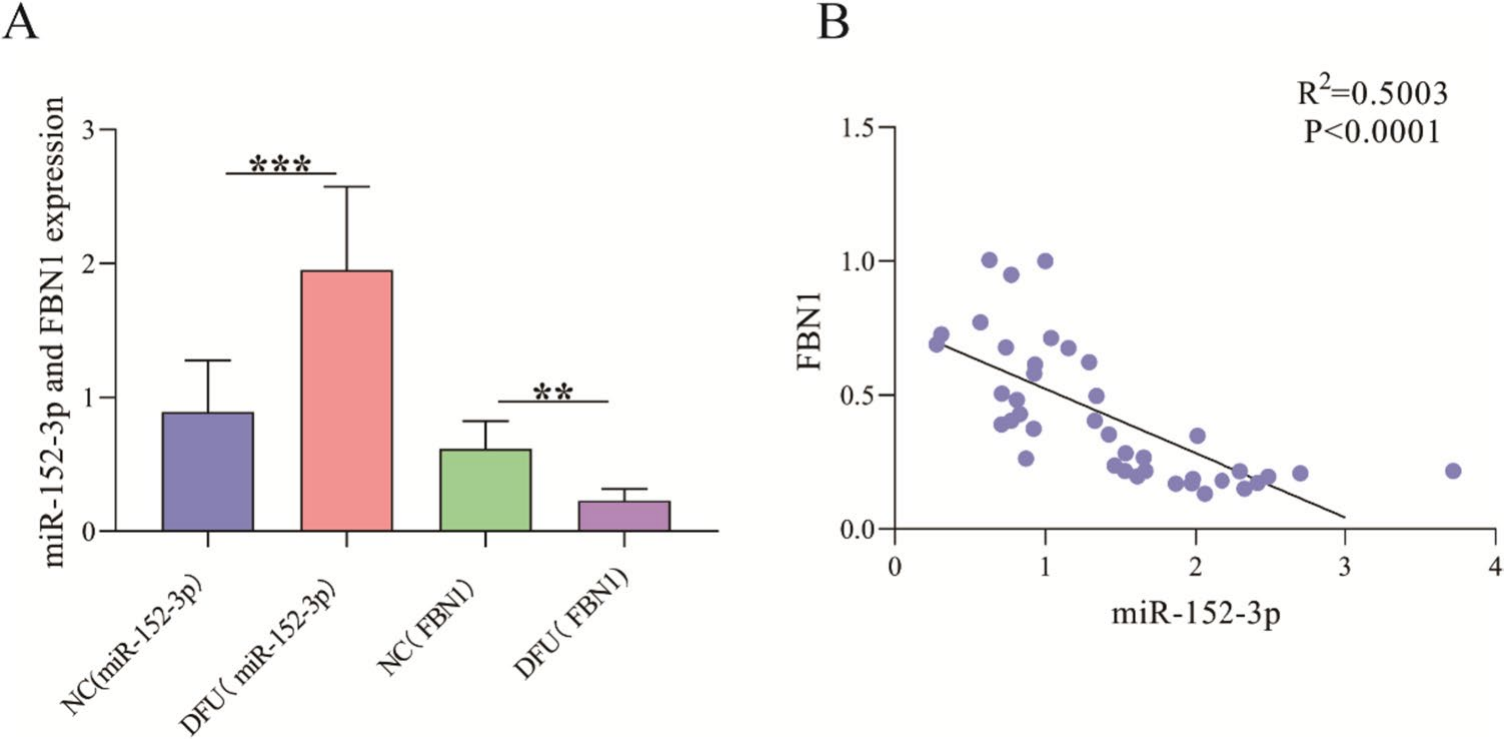
miR-152-3p and FBN1 were abnormally expressed in DFU patients. **A** RT‒qPCR for detecting the levels of miR-152-3p and FBN1; **B** Correlation line plot of miR-152-3p and FBN1. ^**^P < 0.01, ^***^P < 0.001.

We first checked the levels of miR-152-3p and FBN1 in the blood of DFU patients and found that the miR-152-3p level was observably higher than that in healthy individuals, while the level of FBN1 was observably lower than that in healthy individuals (Fig. 1A). miR-152-3p and FBN1 had a linear negative correlation (Fig. 1B).

### Curcumin Attenuates Fibroblast Damage Induced by HG

**Fig. 2:**
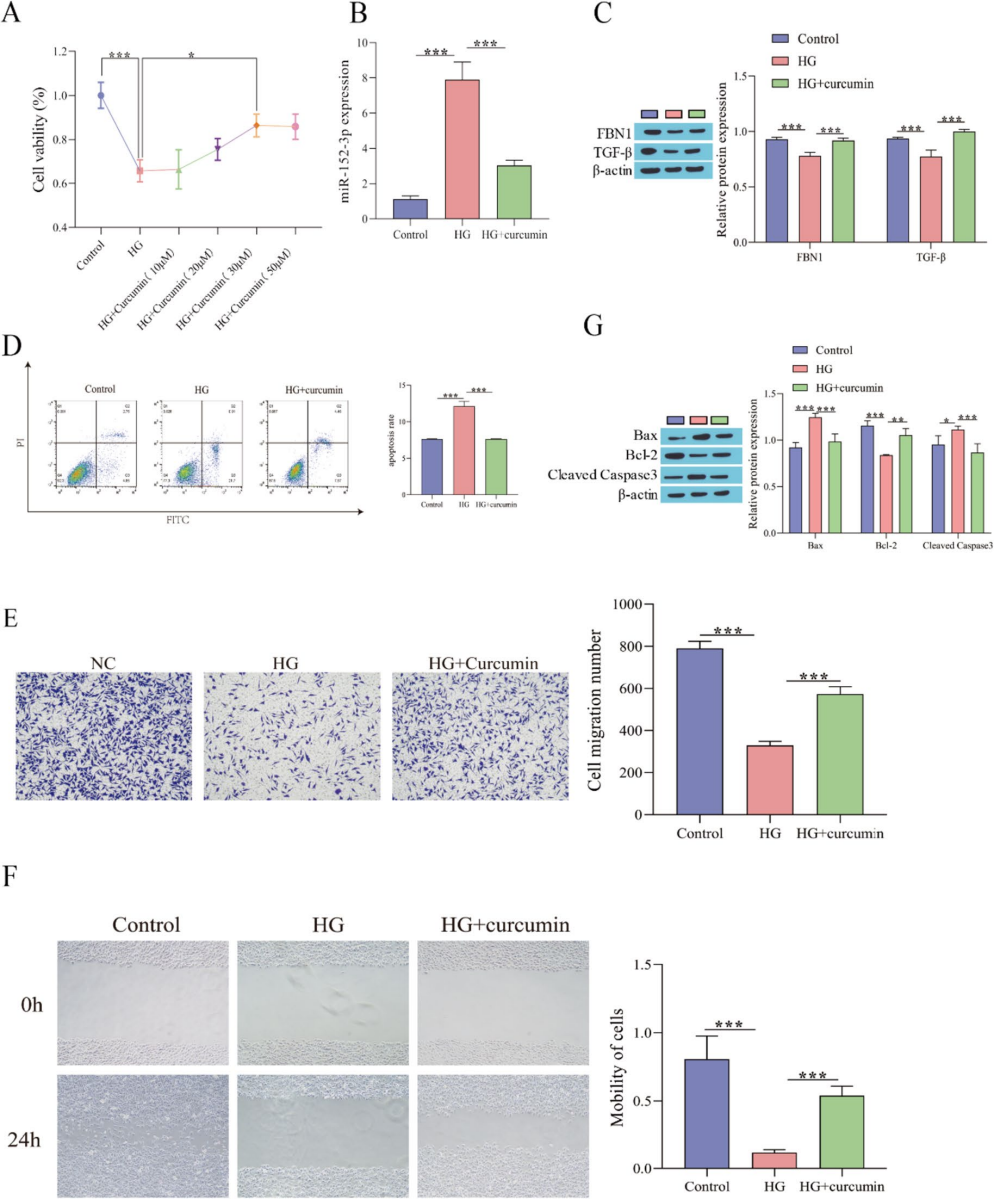
Curcumin alleviates the damage to fibroblasts stimulated by HG. **A** Cell proliferation by CCK-8; **B** RT‒qPCR for detecting miR-152-3p level; **C** Western blot for detecting FBN1 and TGF-β protein level; **D** Flow cytometry for evaluating apoptosis; **E** Transwell for evaluating cell migration; **F** Scratch assay for evaluating cell migration; **G** Western blot for detecting the level of apoptosis-related proteins. ^*^P < 0.05, ^**^P < 0.01, ^***^P < 0.001

To detect the influence of curcumin on fibroblast damage under HG stimulation, we first determined the concentration of curcumin by detecting cell viability and found that compared with the HG group, cell viability was observably enhanced when the curcumin concentration was 30 μM, but there was no prominent alteration in cell viability after continuing to increase the dose (Fig. 2A). Therefore, a concentration of 30 μM was selected for curcumin treatment in subsequent experiments. Then, the detection results of the miR-152-3p level showed that the expression of miR-152-3p was observably elevated after HG treatment, while the level was reduced after adding curcumin (Fig. 2B). Western blot analysis showed that FBN1 and TGF-β expression was observably decreased after HG treatment, while curcumin elevated the levels of FBN1 and TGF-β (Fig. 2C). Apoptosis was detected by flow cytometry and showed that HG treatment facilitated the apoptosis of fibroblasts, and curcumin weakened the influence of HG treatment (Fig. 2D). Transwell and scratch assays were used to detect cell migration, and HG treatment restrained fibroblast migration, while curcumin facilitated fibroblast migration (Fig. 2E, F). Finally, Western blotting detected the expression of apoptosis-related proteins, and the results showed that HG could facilitate the expression of Bax and cleaved caspase-3 and inhibit Bcl-2 expression, while the addition of curcumin weakened the influence of HG treatment (Fig. 2G). These results indicated that curcumin could inhibit the expression of miR-152-3p, promote the expression of FBN1 and TGF-β, inhibit the apoptosis of fibroblasts and promote their migration.

### Curcumin Alleviates the Damage to Fibroblasts Induced by HG by Inhibiting miR-152-3p

**Fig. 3:**
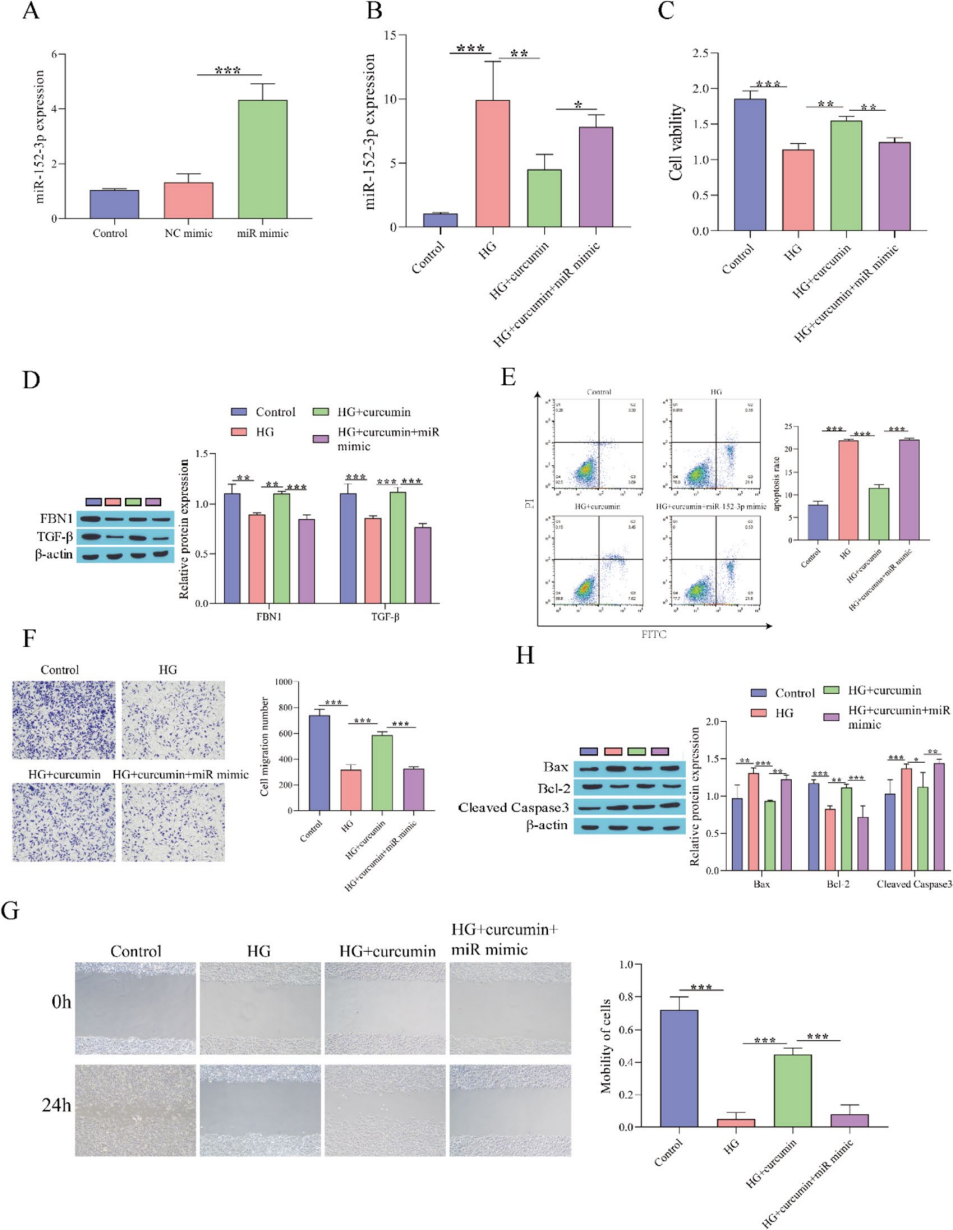
Curcumin alleviates the damage of fibroblasts stimulated by high glucose by restraining miR-152-3p. **A** RT‒qPCR for evaluating the miR-152-3p transfection efficiency; **B** RT‒qPCR for detecting miR-152-3p expression; **C** CCK-8 assay for evaluating cell viability; **D** Western blot for detecting the expression of FBN1 and TGF-β proteins; **E** Flow cytometry for evaluating cell apoptosis; **F** Transwell for detecting cell migration; **G** Scratch assay for detecting cell migration; **H** Western blot for detecting the levels of apoptosis-related proteins. ^*^P < 0.05, ^**^P < 0.01, ^***^P < 0.001. "miR mimic" means "miR-152-3p mimic"

Based on the preceding results, we tested the influence of curcumin on fibroblast damage under high glucose stimulation by regulating miR-152-3p. RT‒qPCR showed that compared with the NC mimic group, the expression of miR-152-3p was significantly upregulated in the miR-152-3p mimic group, indicating successful transfection of the miR-152-3p mimic (Fig. 3A). Compared with the HG group, curcumin treatment inhibited the expression of miR-152-3p, while overexpression of miR-152-3p reversed the effect of curcumin treatment (Fig. 3B). CCK-8 was used to detect cell viability. Cell viability was observably elevated after curcumin treatment compared to the HG group, while the miR-152-3p mimic weakened the effect of curcumin (Fig. 3C). Western blot analysis showed that the levels of FBN1 and TGF-β were inhibited after HG treatment, the levels of FBN1 and TGF-β were elevated after adding curcumin, and overexpression of miR-152-3p further inhibited the levels of FBN1 and TGF-β (Fig. 3D). Flow cytometry to detect apoptosis showed that HG treatment facilitated fibroblast apoptosis, curcumin suppressed fibroblast apoptosis, and overexpression of miR-152-3p facilitated fibroblast apoptosis (Fig. 3E). Next, fibroblast migration was examined, and it was found that curcumin treatment accelerated fibroblast migration compared to the HG group, whereas the miR-152-3p mimic could weakened the influence of curcumin (Fig. 3F, G). Western blot analysis showed that curcumin treatment suppressed the levels of Bax and cleaved caspase3 and accelerated Bcl-2 expression compared to the HG group, while overexpression of miR-152-3p weakened the influence of curcumin (Fig. 3H). These results indicated that curcumin alleviated the damage to fibroblasts stimulated by HG by inhibiting miR-152-3p.

### miR-152-3p Directly Targets FBN1

**Fig. 4:**
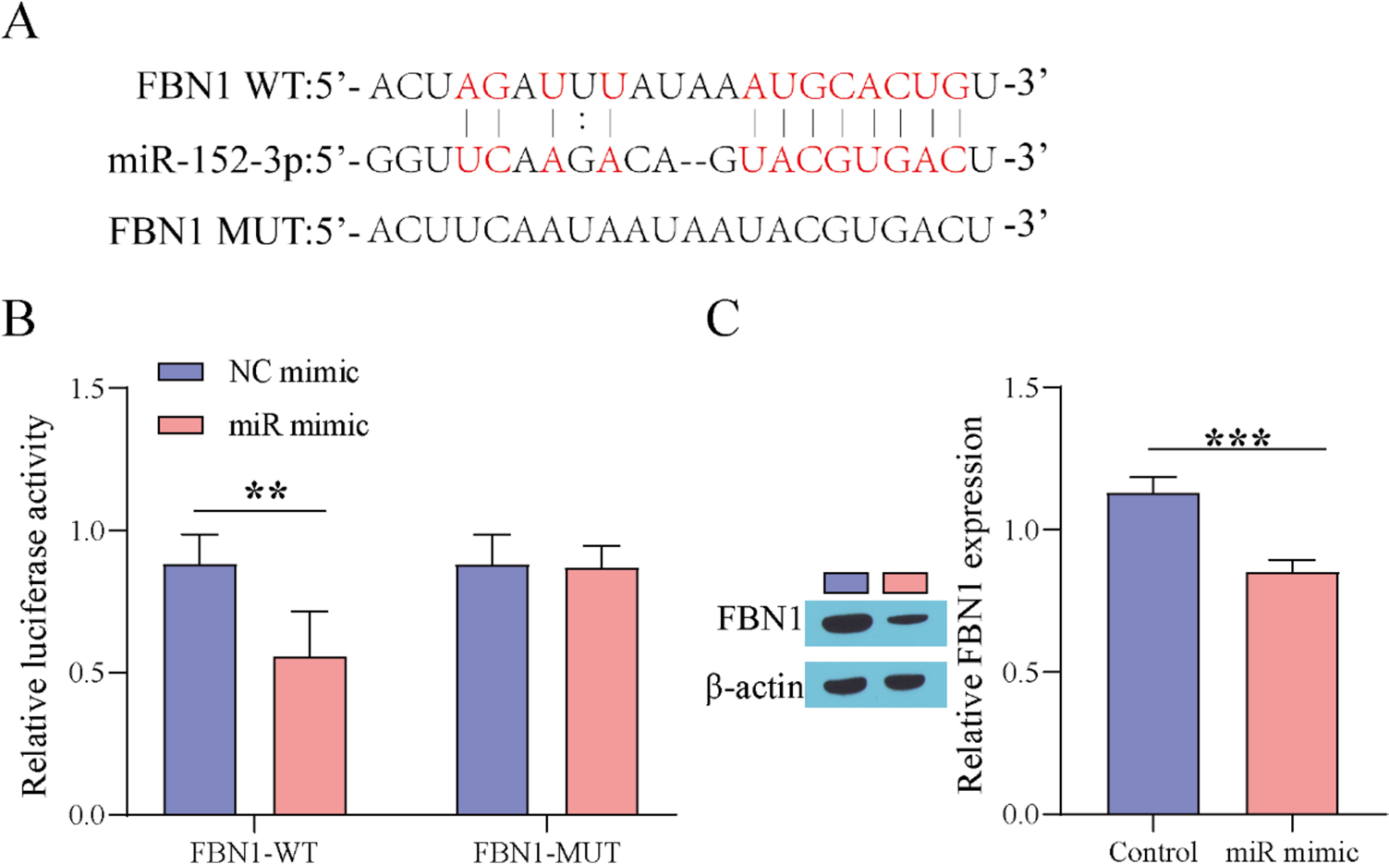
miR-152-3p targets FBN1. **A** Bioinformatics website predicting the targeted binding relationship of miR-152-3p; **B** Dual-luciferase reporter assay; **C** Western blot for detecting FBN1 expression. ^**^P < 0.01, ^***^P < 0.001. "miR mimic" means "miR-152-3p mimic"

To elucidate the downstream regulatory mechanism of miR-152-3p, we used a bioinformatics website (http://starbase.sysu.edu.cn/) to predict the miR-152-3p targeted combination relationship, identifying FBN1 as the downstream target of miR-152-3p (Fig. 4A). It was also certified by a dual-luciferase reporter assay that miR-152-3p could target and bind to FBN1 (Fig. 4B). Finally, Western blot analysis showed that FBN1 expression was prominently decreased after overexpression of miR-152-3p (Fig. 4C). The above results confirm that miR-152-3p targets and negatively regulates FBN1.

### Curcumin Attenuates Fibroblast Injury Induced by HG by Restraining miR-152-3p and Activating the FBN1/TGF-β Pathway

**Fig. 5:**
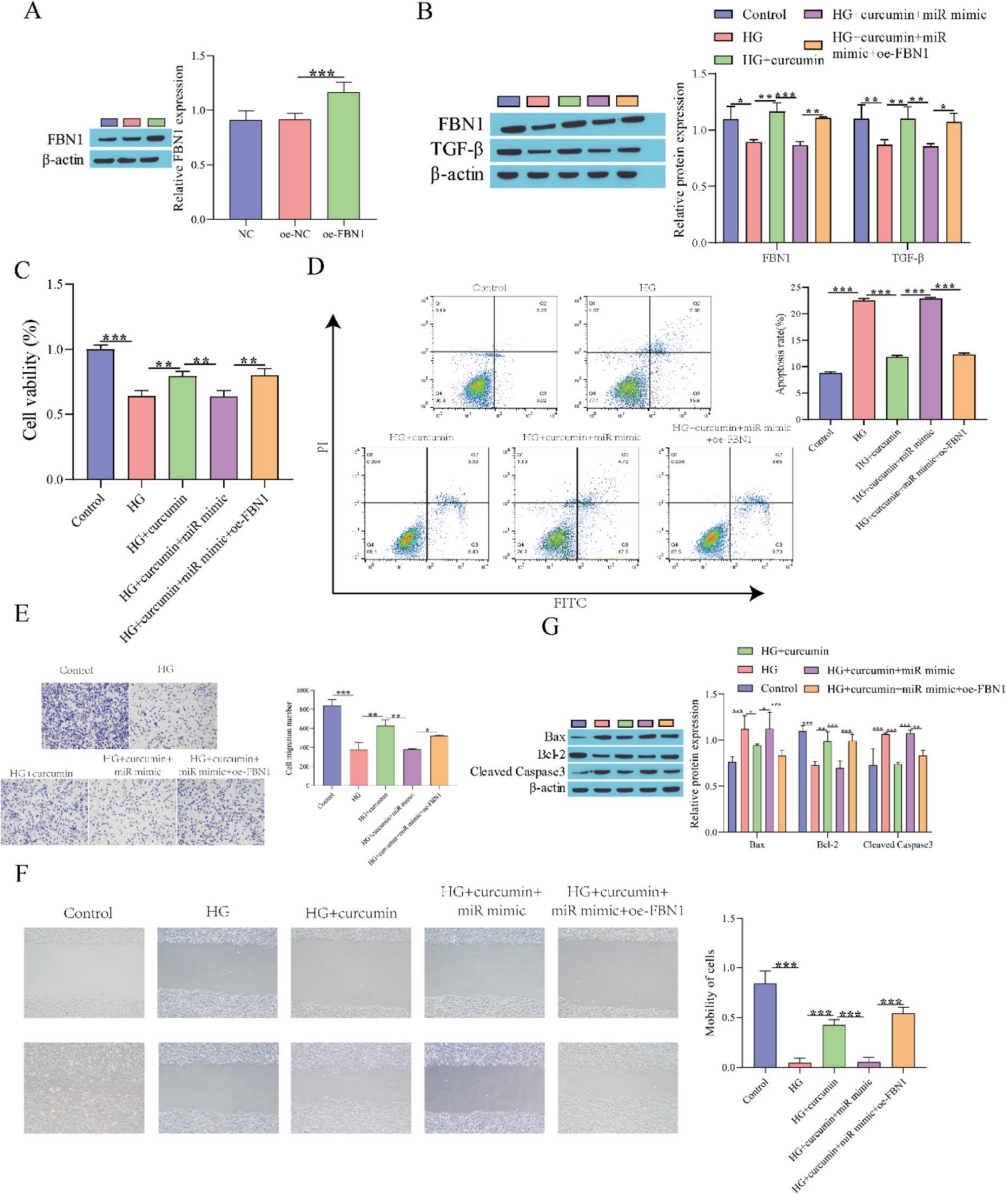
Curcumin alleviates the damage to fibroblasts stimulated by high glucose by inhibiting miR-152-3p and activating the FBN1/TGF-β pathway. **A** Western blot for evaluating FBN1 overexpression efficiency; **B** Western blot for detecting FBN1 and TGF-β proteins expression; **C** CCK-8 assay for evaluating cell viability; **D** Flow cytometry for apoptosis; **E** Transwell for cell migration; **F** Scratch assay for evaluating cell migration; **G** Western blot for detecting apoptosis-related protein expression. ^*^P < 0.05, ^**^P < 0.01, ^***^P < 0.001. "miR mimic" means "miR-152-3p mimic"

The effect of curcumin on HG-induced fibroblast damage through miR-152-3p regulation of the FBN1/TGF-β axis was next examined. First, Western blot for the detection of FBN1 and TGF-β levels showed that compared with the oe-NC group, FBN1 expression was prominently elevated after overexpression of FBN1 (Fig. 5A). Compared with the HG group, FBN1 and TGF-β were prominently elevated after adding curcumin, the effect of curcumin was weakened after overexpressing miR-152-3p, and the influence of the miR-152-3p mimic was reversed after further overexpressing FBN1 (Fig. 5B). Cell viability and apoptosis were measured using CCK-8 and flow cytometry. The results showed that compared with the HG group, cell viability was observably elevated and apoptosis was significantly lessened after curcumin treatment, and the effect of curcumin was weakened after miR-152-3p overexpression. Further overexpression of FBN1 weakened the influence of overexpressing miR-152-3p (Fig. 5C, D). Transwell and scratch assays were used to detect cell migration, and it was found that curcumin treatment facilitated fibroblast migration, while the miR-152-3p mimic could restrain fibroblast migration, and further overexpression of FBN1 facilitated fibroblast migration (Fig. 5E, F). Finally, the expression of apoptosis-related proteins was detected by Western blot and showed that in comparison with the HG group, curcumin treatment diminished the levels of Bax and cleaved caspase3 and elevated Bcl-2 levels, while the miR-152-3p mimic weakened the influence of curcumin. Further overexpression of FBN1 weakened the influence of the miR-152-3p mimic (Fig. 5G). In conclusion, overexpression of miR-152-3p can weaken the influence of curcumin on fibroblasts, and overexpression of FBN1 can also weaken the impact of miR-152-3p on fibroblasts, indicating that curcumin can reduce the damage of fibroblasts stimulated by high glucose by inhibiting miR-152-3p and activating the FBN1/TGF-β pathway.

### Curcumin Accelerates Wound Healing in DFU Rats by Inhibiting miR-152-3p and Activating the FBN1/TGF-β Pathway

**Fig. 6:**
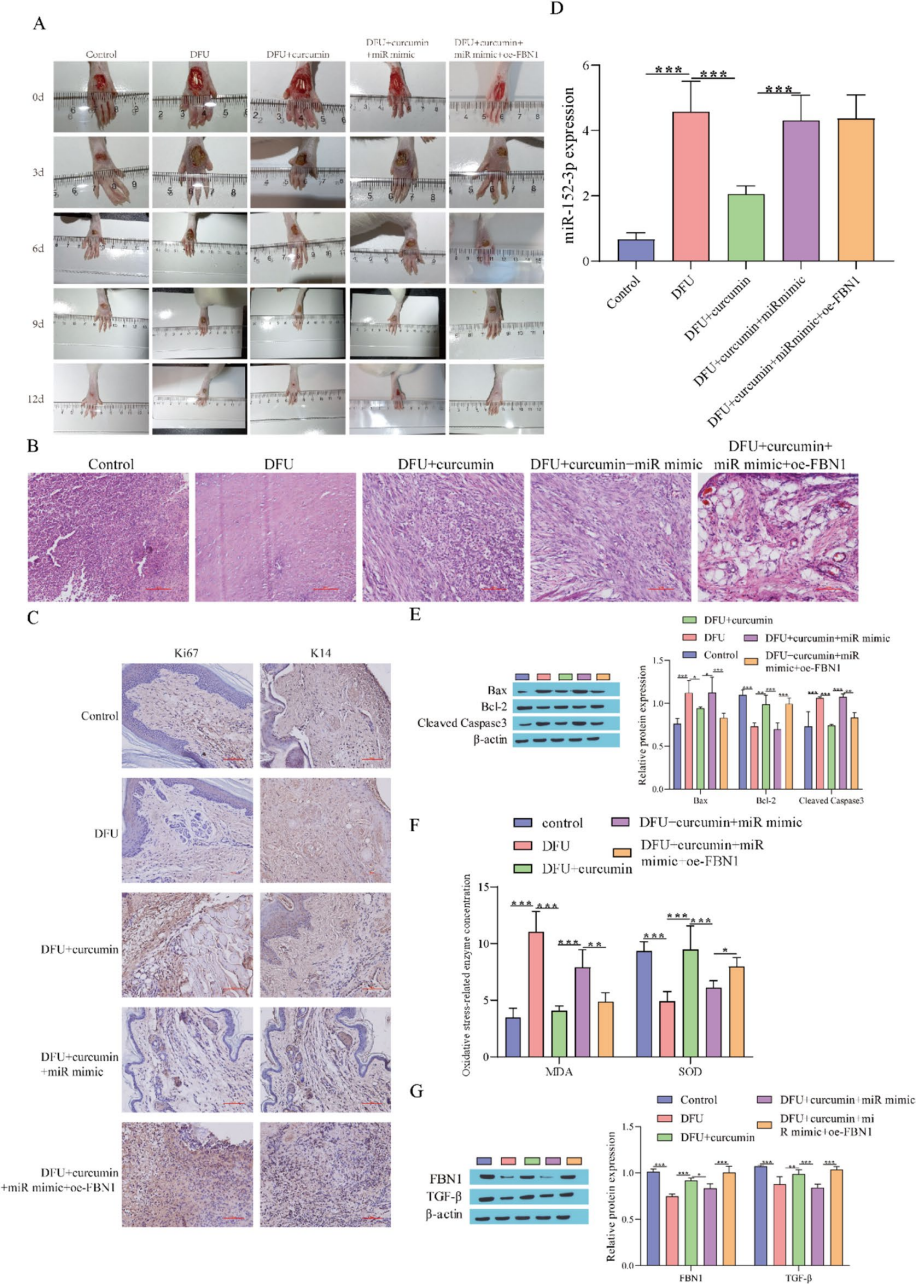
Curcumin accelerates wound healing in DFU rats by inhibiting miR-152-3p and activating the FBN1/TGF-β pathway. **A** Degree of foot wound healing in rats; **B** HE staining was used to observe the pathological changes; **C** The expression levels of Ki67 and K14 were detected by immunohistochemical staining; **D** RT‒qPCR for detecting miR-152-3p expression; **E** Western blot for detecting apoptosis-related protein levels; (F) MDA and SOD were detected by kits; **G** Western blot for detecting FBN1 and TGF-β protein levels. ^*^P < 0.05, ^**^P < 0.01, ^***^P < 0.001. "miR mimic" means "miR-152-3p mimic"

To improve the scientific validity of the experiment, we verified the relevant mechanism at the animal level. Wound healing in rats was first observed, and it was found that curcumin accelerated wound recovery in DFU rats, and overexpression of miR-152-3p suppressed curcumin-mediated wound healing, while the suppressive effect from overexpressed miR-152-3p was weakened by overexpression of FBN1 (Fig. 6A). The HE staining results showed that in comparison with the control group, the angiogenesis capacity of the DFU group was observably lessened, curcumin treatment promoted angiogenesis, and overexpression of miR-152-3p could inhibit angiogenesis, while overexpression of FBN1 reversed the inhibitory effect of overexpression of miR-152-3p (Fig. 6B). Immunohistochemical analysis showed that the levels of Ki67 and K14 in the DFU group were observably lower than those in the control group. Curcumin treatment promoted the expression of Ki67 and K14, and the miR-152-3p mimic reversed the effect of curcumin, while overexpression of FBN1 reversed the inhibitory effect of the miR-152-3p mimic (Fig. 6C). Compared with the DFU group, curcumin treatment diminished miR-152-3p expression, and the miR-152-3p mimic could facilitate miR-152-3p expression, while overexpression of FBN1 resulted in no significant changes (Fig. 6D). Western blot analysis showed that in comparison with the DFU group, curcumin treatment decreased the levels of Bax and cleaved caspase-3 and increased the Bcl-2 level, while miR-152-3p overexpression reversed the effect of curcumin. Further overexpression of FBN1 reversed the effect of overexpression of miR-152-3p (Fig. 6E). Then, the changes in MDA and SOD were detected, and the results showed that compared to the control group, the content of MDA in the DFU group was observably elevated, but the content of SOD was observably lessened, the treatment of curcumin observably lessened the content of MDA and elevated the level of SOD, and the overexpression of miR-152-3p reversed the influence of curcumin. Further overexpression of FBN1 reversed the effect of overexpressing miR-152-3p (Fig. 6F). Finally, Western blot analysis showed that in comparison with the DFU group, curcumin treatment increased the levels of FBN1 and TGF-β, and the miR-152-3p mimic weakened the effect of curcumin, while overexpression of FBN1 weakened the suppressive effect of the miR-152-3p mimic (Fig. 6G). In summary, curcumin accelerates wound healing in DFU rats by inhibiting miR-152-3p and activating the FBN1/TGF-β pathway.

## Discussion

DFU affects approximately 18.6 million people worldwide each year and is associated with increased rates of amputation, which increases the risk of death [27]. Studies have shown that in diabetic patients with foot wounds, strict blood sugar control reduces the chance of amputation [28]. In addition, DFU status is worsened by peripheral vascular disease and decreased blood flow. Currently, DFU requires a multimodal treatment strategy that includes controlling blood sugar, eradicating infections, promoting ulcer healing, and relieving high pressure [29]. However, in human and animal studies, there is little information on the influences of vitamin and inartificial ingredient replenishment on wound healing and DFU metabolism [30]. Curcumin is a naturally occurring low molecular weight polyphenolic component found in the rhizome of turmeric [31]. Over the years, curcumin has been used as a traditional remedy for inflammation and wound healing, and it works at diverse stages, such as inflammation, maturation, and proliferation, thereby accelerating the entire process of wound healing [29]. Studies have shown that curcumin treatment can cause fibroblasts to infiltrate into the wound site and accelerate wound healing [32]. In addition, curcumin has been found to be beneficial for DFU and may be a potential candidate for treatment [33]. Therefore, it is important to explore the specific molecular mechanism of curcumin's role in DFU. We induced fibroblast injury by HG, and curcumin inhibited the apoptosis of fibroblasts, promoted their migration ability, and alleviated the damage of fibroblasts stimulated by HG. Furthermore, curcumin treatment promoted angiogenesis and accelerated wound healing in DFU rats. This suggests that curcumin plays an important role in the process of alleviating DFU.

According to previous studies, there are many miRNAs involved in the treatment of different diseases by curcumin. Studies have shown that curcumin can attenuate vascular calcification through the exosomal miR-4b-92p/KLF3 axis [34], and curcumin therapy protects PC12 cells from high glucose-induced inflammatory responses by promoting the expression of miR-218-5p [35]. In this study, we found that miR-152-3p was highly expressed in DFU patients. Moreover, studies have shown that inhibiting the expression of miR-152-3p can promote DFU wound healing [36]. Therefore, hyperglycemia may adversely affect DFU by altering the expression of miR-152-3p. Then, we investigated the effect of curcumin on the progression of DFU by influencing the expression of miR-152-3p. The results showed that in HG-stimulated fibroblasts and DFU rat models, the expression of miR-152-3p was decreased after curcumin treatment, and overexpression of miR-152-3p weakened the protective effect of curcumin on HG-induced fibroblasts and inhibited wound healing in DFU rats, suggesting that curcumin alleviates the progression of DFU by inhibiting miR-152-3p.

In this study, the downstream regulatory mechanism of miR-152-3p was further explored. Based on bioinformatics prediction and double luciferase gene reporting experiments, FBN1 was identified as the downstream target of miR-152-3p. FBN1 was found to regulate the bioavailability of TGF-β [37]. Li et al. [20] have shown that suppression of miR-29b can elevate FBN1 expression, thereby facilitating fibroblast proliferation and migration, restraining apoptosis, and ultimately accelerating wound healing in DFUs. As a pleiotropic growth factor in wound healing, TGF-β plays a momentous role in immune regulation, extracellular matrix production, proliferation and differentiation. High expression of TGF-β has been reported to stimulate myofibroblast differentiation in vivo and in vitro [38]. Mao [39] reported that upregulation of TGF-β can accelerate wound healing in DFU rats. Therefore, in this study, we investigated the effect of miR-152-3p on DFU by regulating the FBN1/TGF-β pathway. The results showed that overexpression of miR-152-3p could inhibit the expression of FBN1 and TGF-β, and in HG-stimulated fibroblasts and DFU rat models, overexpression of FBN1 could weaken the effect of miR-152-3p, thereby promoting the protective effect of curcumin on HG-induced fibroblasts and promoting wound healing in DFU rats. This suggests that miR-152-3p promotes the progression of DFU by inhibiting the expression of FBN1 and TGF-β.

In summary, curcumin activates the FBN1/TGF-β pathway by inhibiting miR-152-3p, thereby inhibiting HG-induced fibroblast apoptosis, promoting fibroblast proliferation and migration, alleviating HG-induced fibroblast damage, and promoting angiogenesis in DFU rats, thereby accelerating wound healing in DFU rats. Our results suggest that the miR-152-3p/FBN1/TGF-β signaling pathway plays an important role in wound healing in DFU. This provides a new theoretical basis for curcumin treatment of DFU and may become a potential therapeutic target for DFU. However, there are some limitations in our study. We only observed this phenomenon through cell and animal experiments, and there is a lack of clinical trials. To clinically apply curcumin for the treatment of DFU, further research is needed in the future.

## Data Availability

The datasets used and/or analyzed during the current study are available from the corresponding author upon reasonable request.
